# A cross-national study on gender differences in suicide intent

**DOI:** 10.1186/s12888-017-1398-8

**Published:** 2017-06-29

**Authors:** Aislinné Freeman, Roland Mergl, Elisabeth Kohls, András Székely, Ricardo Gusmao, Ella Arensman, Nicole Koburger, Ulrich Hegerl, Christine Rummel-Kluge

**Affiliations:** 1 Klinik und Poliklinik für Psychiatrie und Psychotherapie der Universität Leipzig, Semmelweisstraße 10, Haus 13, 04103 Leipzig, Germany; 2 Semmelweis University, Budapest, Hungary; 3 New University of Lisbon, Lisbon, Portugal; 4 National Suicide Research Foundation & Department of Epidemiology and Public Health, University College Cork, Cork, Ireland; 5 Forschungszentrum Depression der Stiftung Deutsche Depressionshilfe, Leipzig, Germany

**Keywords:** Suicide, Attempt, Intent, Gender differences, Suicidal behaviour

## Abstract

**Background:**

Suicide accounts for over 58,000 deaths in Europe per annum, where suicide attempts are estimated to be 20 times higher. Males have been found to have a disproportionately lower rate of suicide attempts and an excessively higher rate of suicides compared to females. The gender difference in suicide intent is postulated to contribute towards this gender imbalance. The aim of this study is to explore gender differences in suicide intent in a cross-national study of suicide attempts. The secondary aims are to investigate the gender differences in suicide attempt across age and country.

**Methods:**

Data on suicide attempts (acquired from the EU-funded OSPI-Europe project) was obtained from eight regions in Germany, Hungary, Ireland and Portugal. Suicide intent data was categorized into ‘Non-habitual Deliberate Self-Harm’ (DSH), ‘Parasuicidal Pause’ (SP), ‘Parasuicidal Gesture’ (SG), and ‘Serious Suicide Attempt’ (SSA), applying the Feuerlein scale. Gender differences in intent were explored for significance by using χ^2^-tests, odds ratios, and regression analyses.

**Results:**

Suicide intent data from 5212 participants was included in the analysis. A significant association between suicide intent and gender was found, where ‘Serious Suicide Attempts’ (SSA) were rated significantly more frequently in males than females (*p* < .001). There was a statistically significant gender difference in intent and age groups (*p* < .001) and between countries (*p* < .001). Furthermore, within the most utilised method, intentional drug overdose, ‘Serious Suicide Attempt’ (SSA) was rated significantly more often for males than females (*p* < .005).

**Conclusions:**

Considering the differences in suicidal intent between males and females highlighted by the current study, gender targeted prevention and intervention strategies would be recommended.

## Background

Suicidal behaviour is a significant public health problem. Suicide is the 13th leading cause of death globally [1] and accounts for over 58,000 deaths in Europe a year [2]. Suicide appears to be a male phenomenon, as death rates from suicide are four-to-five times higher for men than for women across the European Union [3].

For suicide attempts, for which the rate is estimated to be 20 times higher than that of suicides [4], the gender gap is less pronounced, with females demonstrating a disproportionately higher rate of suicide attempts compared to males [5]. The Male:Female ratio of age-standardized suicide rates globally is 1.9 [4]. This phenomenon of men completing suicide more frequently than females, while females engage significantly more frequently in suicide attempts, is known as the gender paradox of suicidal behaviour [6, 7].

Many studies have sought to explain the gender gap in suicidal behaviour by addressing lethality, suggesting that females survive suicide attempts more often than males because they use less lethal means [8, 9], and their outcomes are less lethal compared to males even when using the same method [8]. Major Depression (which is approximately twice as common in females, and is known to underlie more than half of all suicides) has also been proposed to account for a higher incidence of suicidal behaviours in females [10, 11]. This could be a contributing factor to the lower rates of suicidal behaviour in males overall, however, this does not account for the excessive rate of completed male suicides compared to female suicides. Psychosocial risk factors have also been found to contribute to the discrepancy of rates between male and female suicidal behaviour, where unemployment, retirement and being single were all significant risk factors for suicide in males, whereas no significant risk factors other than mental illness were reported for females [11, 12]. So far, studies aimed to disentangle the gender gap have reported inconsistent findings; therefore, suicide intent has been at the forefront of suicide research in order to contribute to the explanation of this gender imbalance.

Suicide intent in this context is characterised as “an individual’s desire to bring about his or her own death” [13], which specifically excludes motives for attempting suicide. Studies exploring intent have found that the type of suicide intent at the time of a suicide attempt is associated with an elevated risk of completed suicide [14, 15], which is particularly prudent within the female population, where the association between the type of suicidal intent and completed suicide is markedly higher [15].

To date, there have been some studies that have investigated this relationship between suicidal intent and gender. Theorists investigating suicide intent argue that the excess rate of attempted suicide in females, plus the stronger association between suicide attempts and death in males, is indicative of a stronger degree of intent to die in males than females [16, 17]. Some studies addressing gender differences in intent have reported no significant differences between males and females [6, 18–20] while others revealed significant associations between suicide intent and gender [15, 21, 22]. Although previous studies have documented gender differences in suicide intent, the findings need to be interpreted in light of methodological issues such as small sample sizes, absence of consistent operational terms of suicide intent and the assessment of intent and motives as a single concept. In order to fully comprehend the gender paradox in suicidal behaviour, further research to explicate this gender gap in suicide intent remains to be explored. The present study aims to overcome the methodological issues from previous research by utilising both standardised definitions and a large database retrieved cross-nationally for increased validity and effect.

### Study aims

The main aim of the current study is to bring clarity to previous studies in this space, and to shed more light on the role of gender differences in suicide intent. The secondary aims are to investigate the gender differences in suicide attempt across age and country. Our main hypothesis is that male suicide attempts will be rated more frequently as a serious suicide attempt overall than female ones. Additionally, we expect that within each age group, country, and most utilised suicide method, males will be rated more frequently in the serious suicide attempt group compared to females.

## Methods

### Data collection

Data on suicide attempts was obtained from the EU-funded OSPI-Europe project (“Optimising Suicide Prevention Programmes and their Implementation in Europe”) [23] from eight model regions in Europe (Ireland, Hungary, Germany and Portugal). This project was conducted from 2008 to 2013, and its aim was to develop and evaluate an optimised version of the 4-level intervention programme of the EAAD (European Alliance Against Depression) [24, 25] targeting depression and suicidal behaviour. Attempted and completed suicides were assessed as the primary outcome parameter, where adults who attempted suicide were included in the study, and those who engaged in habitual self-harm were excluded. Project results have been reported elsewhere [9, 26, 27].

The data for the current study were obtained from the eight regions in the four participating countries, where each region adhered to the following standardised definition of Suicide Attempt: “an act with a non-fatal outcome in which an individual deliberately initiates a non-habitual behaviour that, without intervention from others, will cause a self-harm, or deliberately ingests a substance in excess of the prescribed or generally recognised therapeutic dosage, and which is aimed at realising changes which the subject desired, via the actual or expected physical consequences” [28]. Suicidal behaviour elsewhere referred to as parasuicide [29] is included, while habitual intentional self-harm is excluded from this definition. A standardised questionnaire for the registration of suicide attempts and a codebook listing the associated variables (e.g. gender [in this context, defined as ‘the state of being male or female], age and country of suicide attempter, intent and method of suicide attempt) was employed by all eight regions to ensure comparability and consistency in the standardisation of data collection. The specific time and method of data collection was dependant on the local circumstances of the participating centres (see Table 1).

**Table 1:** The data collection procedures of the participating countries

Country	Data collection procedure
Germany	• Collection period: June 2008 to May 2011 • 2 centres registered the data via personal interviews of the patients after suicide attempt • 2 centres retrospectively assessed patient records
Ireland	• Collection period: April 2009 to March 2012 • Systematically registered cases of suicide attempts presenting to two hospital emergency departments • Data from retrospective assessment of patient records
Hungary	• Collection period: January 2009 to December 2011 • Data from one hospital analyzed retrospective assessment of patient records
Portugal	• Collection period: April 2009 to March 2012 • Data from one hospital analyzed retrospective assessment of patient records

Table 1 summarises the data collection procedures in the participating countries.

Data on type of suicide intent (outlined below) were rated based on the judgement of the clinical staff. All personnel involved in the eight assessment regions were trained in administering the measurement and in how to proceed in unclear cases.

### Definition of variables

**Fig. 1:**
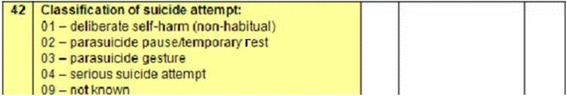
Feuerline Scale

Suicide intent was assessed as the dependent variable. The type of suicide intent was classified by a clinical staff member based on the nature of the suicide attempt using the Feuerlein Scale. The Feuerlein Scale [30] (see Fig. 1 for the format the scale had in the standardised questionnaire) is a categorical, non-ordinal based evaluation tool which was developed in order to classify different psychological intentions for suicidal acts based on the circumstances of the patients’ suicidal act, and has four categories: 1) (non-habitual) Deliberate Self-Harm (DSH); 2) Parasuicidal Pause (SP)- refers to suicidal behaviour carried out mainly to escape from an unbearable situation/from problems; 3) Parasuicidal Gesture (SG) – refers to an appellative or manipulative suicidal act (and excludes ideas or threats without any action performed); and 4) Serious Suicide Attempt (SSA) – refers to suicidal behaviour carried out with a clear intent to die [30].

The following independent variables were also addressed: gender, age, country and method of suicide attempt.

For age, means and standard deviations were computed, and age groups were aggregated in order to compare the younger age group as the reference category (< 30 years) with a middle (30–45 years) and higher age group (> 45 years).

### Data analyses

Gender differences in the distribution of suicide intent were tested for significance by using the χ^2^-test for two-by-four tables. These analyses were repeated, using the factors “country” and “age group” as strata. Standardized residuals were computed in order to identify those cells in the cross tables which added the most to the statistically significant results of the corresponding χ^2^-tests. The effect of age group on suicide intent within each gender was also analysed using χ^2^-tests. In addtion, a χ^2^ analysis for suicide methods was calculated to identify types of suicide intent and their association with gender.

The hypothesis whether several independent variables (gender, age group, country) had a separate influence on the category of suicide intent regarding the frequency of suicide attempts was tested by running four stepwise binary logistic regressions. The binary dependent variable was each category of suicide intent (Deliberate self-harm: 1 = Yes; 0 = No; Parasuicidal Pause: 1 = Yes; 2 = No; Parasuicidal Gesture: 1 = Yes; 2 = No; Serious Suicide Attempt: 1 = Yes; 2 = No). For quantifying the strength of associations between the independent variables and gender, odds ratios (OR) and the corresponding 95% confidence intervals (CI) were applied.

Moreover, in order to answer the question whether gender differences in suicide intent were age-dependent or not, a multinomial regression analysis was used to investigate the interaction of the factors “gender” and “age group” regarding suicide intent as a dependent variable.

The statistical tests were two-tailed and performed using the statistical software package for IBM SPSS Statistics 20™ for Windows (IBM, New York, USA). The significance level was set at .05.

## Results

In total, 8189 suicide attempts were registered, however, the final sample consisted of 5212 subjects (63.65% of the complete OSPI-Europe sample), with 52.1% of the attempted suicides rated as a Serious Suicide Attempt (SSA), 20.6% as a Parasuidal Gesture (SG), 14.7% as a Parasuicidal Pause (SP) and 12.7% as Deliberate Self-Harm (DSH). 40.6% of the sample were males and 59.4% females, with a mean age of 39.16 years. Additionally, 25.0% of the sample were from Germany, 19.3% were recorded from Hungary, 29.5% from Ireland and 26.2% were from Portugal. 67.9% of the final sample attempted suicide by intentional drug overdose, 7.6% intentional self-poisoning by other means, and 3.5% by hanging.

### Suicide intent and gender

The association between suicide intent and gender was statistically significant, *X* ^2^ (3, *N* = 5212) = 39.94; *p* < .001. According to the standardized residuals, SG and SSA contributed most to this significant difference: females were rated significantly more frequently in SP and SG than males, whereas SSA were rated significantly more often in males than females (see Table 2). There was no significant difference in the frequency of suicide attempts rated as DSH between males and females.

**Table 2:** Types of suicide intent and their association with gender, countries and age

		Gender	Deliberate Self-Harm (DSH) [%](SR)	Para-suicidal Pause (SP) [%](SR)	Para-suicidal Gesture (SG) [%](SR)	Serious Suicide Attempt (SSA) [%](SR)	*p* value	Total
Gender (*n* = 5212)		Male	260 [12.3%]	281 [13.3%]*	367 [17.4%]***	1206 [57%]***	< .001^a^	2114
		Female	402 [13%]	484 [15.6%]	705 [22.8%]	1507 [48.6%]		3098
Country	Germany (*n* = 1306)	Male	16 [2.9%]	56 [10.2%]	112 [20.4%]	366 [66.5%]	= .37^a^	550
		Female	23 [3%]	97 [12.8%]	165 [21.8%]	471 [62.3%]		756
	Hungary (*n* = 1004)	Male	78 [18.1%]***	146 [33.8%]**	19 [4.4%]	189 [43.8%]	= .002^a^	432
		Female	61 [10.7%]***	241 [42.1%]**	20 [3.5%]	250 [43.7%]		572
	Ireland (*n* = 1537)	Male	57 [7.9%]	57 [7.9%]	166 [22.9%]**	446 [61.4%]**	= .007^a^	726
		Female	73 [9%]	62 [7.6%]	243 [30%]**	433 [53.4%]**		811
	Portugal (*n* = 1365)	Male	109 [26.8%]	22 [5.4%]*	70 [17.2%]***	205 [50.5%]***	< .001^a^	406
		Female	245 [25.5%]	84 [8.8%]*	277 [28.9%]***	353 [36.8%]***		959
Age Group	<30 years (*n* = 1810)	Male	81 [11.1%]	92 [12.6%]	161 [22.1%]*	394 [54.1%]***	= .005^a^	728
		Female	149 [13.8%]	156 [14.4%]	284 [26.2%]*	493 [45.6%]***		1082
	30–45 years (*n* = 1695)	Male	95 [13.2%]^+^	103 [14.3%]*	113 [15.7%]**	410 [56.9%]***	<.001^a^	721
		Female	160 [16.4%]^+^	177 [18.2%]*	208 [21.4%]**	429 [44%]***		974
	>45 years (*n* = 1707)	Male	84 [12.6%]*	86 [12.9%]	93 [14%]**	402 [60.5%]	<.001^a^	665
		Female	93 [8.9%]*	151 [14.5%]	213 [20.4%]**	585 [56.1%]		1042

Included cases refer to persons with valid gender data who had not changed their gender

*M* arithmetic means, *n* sample size, *p* significance level, *SD* standard deviation, *sr* standardized residual

^+^ *p* < .10; *p < .05; **p < .01; ***p < .001 (refers to the *p* values of the cross-tables)

^a^According to the results of a X^2^-test

### Suicide intent, gender and age

Overall, there were no significant gender differences in age (mean age (S.D.) in males: 38.77 (16.17) years; mean age (S.D.) in females: 39.42 (17.51) years; Z = −0.52; *p* = 0.60 (Mann-Whitney U test)). According to the Mann-Whitney U Tests, there was a significant difference in age between males and females in the DSH group, where the mean age for males was significantly higher than in females (39.93 > 36.99, *p* = 0.015).

Within the youngest age group (<30 years), there was a significant association between SG and SSA with gender. In this age group, male suicide attempts were rated as SSA significantly more often than female suicide attempts, while SG were rated significantly more times for females than males. In the middle age group (30–45 years), female suicide attempts were rated as SP and SG significantly more frequently than male suicide attempts, while male suicide attempts were rated as SSA significantly more than female suicide attempts. In the older age group (>45 years), female suicide attempts were rated as SG significantly more frequently than male suicide attempts, while male suicide attempts were rated as DSH significantly more than female suicide attempts.

A multinomial regression analysis was used to investigate the interaction of the variables “gender” and “age group”, with suicide intent as the dependent variable. A significant interaction effect was obtained, *X* ^*2*^ (6, *n* = 5212) = 15.96; *p* = .014. This finding represents an age-dependency of gender differences in suicide intent.

### Suicide intent, gender and country

**Fig. 2:**
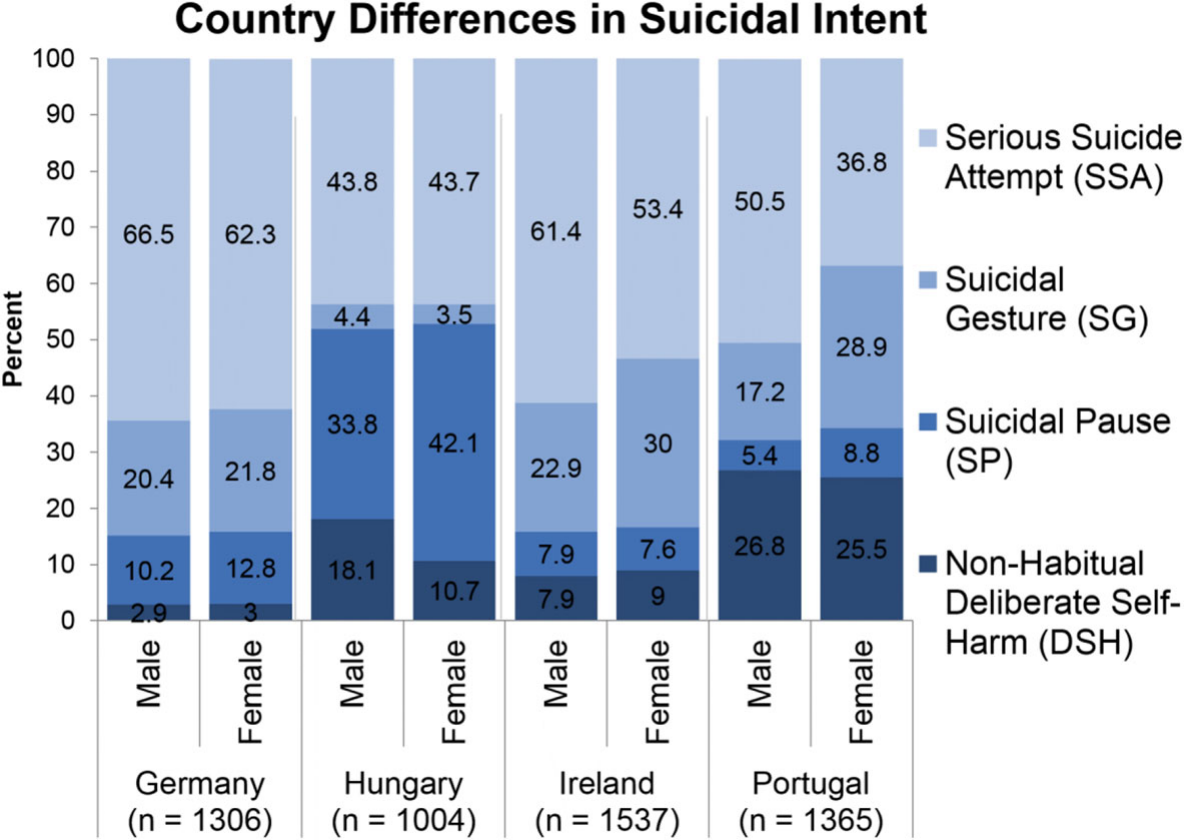
Country differences in suicide intent

There was a statistically significant association between suicide intent and country both in males, *X* ^*2*^ (9, *N* = 5212) = 393.29; *p* < .001, and females, *X* ^*2*^ = (9, *N* = 5212) = 700.64; *p* < .001 [see Fig. 2]. Hungary, *X* ^*2*^ (3, *N* = 5212) = 14.66; *p* = .002, Ireland, X^2^ = (3, *N* = 5212) = 12.21; *p* = .007 and Portugal, *X* ^*2*^ (3, *N* = 5212) = 32.56; *p* < .001, all reported significant differences between males and females in suicide intent. There was no significant difference between the genders in suicide intent in Germany (*p* = .37). In Hungary, a significant difference between the genders was found in DSH and SP, where male suicide attempts were rated as DSH significantly more often than female suicide attempts, and female suicide attempts were rated as SP significantly more than male attempts. In Ireland and Portugal, significant differences in intent between males and females were reported, where female suicide attempts were rated significantly more frequently as SG and significantly less frequently as SSA than males.

### Analyses for different suicide methods

In order to address the question of whether the method of suicide attempt plays a role in the association between suicide intent and gender, a Chi-square analysis was performed. In terms of the three most frequently used methods of suicide attempts (intentional drug overdose, intentional self-poisoning by other means, and hanging), there was only a significant difference in suicide intent and gender within the intentional drug overdose method (*p* = .0041). Of those who chose this method, males were rated as SSA significantly more frequently and as DSH and SG significantly less frequently than females (see Table 3).

**Table 3:** Results of a χ^2^ analysis for suicide methods identifying types of suicide intent and their association with gender

	Gender	Non-Habitual Deliberate Self-Harm (DSH) [%](SR)	Para-suicidal Pause (SP) [%](SR)	Para-suicidal Gesture (SG) [%](SR)	Serious Suicide Attempt (SSA) [%](SR)	*P* value	Total
Intentional drug overdose *n* = 3542	Male	116 [9.5%]*	210 [17.3%]	239 [19.7%]*	650 [53.5%]**	.004^a^	1215
	Female	275 [11.8%]*	404 [17.4%]	537 [23.1%]*	1111 [47.7%]**		2327
Intentional self-poisoning by other means *n* = 395	Male	18 [8.7%]	21 [10.1%]	40 [19.2%]	129 [62.0%]	.403^a^	208
	Female	12 [6.4%]	22 [11.8%]	47 [25.1%]	106 [56.7%]		187
Hanging *n* = 181	Male	2 [1.6%]	10 [7.9%]	13 [10.2%]	102 [80.3%]	.944^a^	127
	Female	1 [1.9%]	3 [5.6%]	5 [9.3%]	45 [83.3%]		54

Included cases refer to persons with valid gender data who had not changed their gender

*p* significance level, *SR* standardized residual

^+^ *p* < .10; *p < .05; **p < .01; ***p < .001

^a^According to the results of a X^2^-test

### Results of the binary logistic regression analyses

Table 4 displays the results of the binary logistic regression analyses which investigated the impact of age, gender and country on each category of suicide intent. The analyses explained between 7.1–16.5% of the total variance which suggests that there are other confounding variables that account for the variance in suicide intent and gender.

**Table 4:** Results for binary logistic regression analyses of variables influencing the type of suicide intent according to the Feuerlein Scale

Type of suicide intent	Independent variables	Odds ratio (OR)	95% CI for the OR	*p*	Nakelkerke’s R^2^
Non-habitual deliberate self- harm	Male gender^a^	.78	.53–1.15	*p =* 0.21	.14
	Age group^b^	---	---	*p* < .001	---
	- 30-45 years	.89	.69–1.16	*p* = 0.39	---
	- >45 years	.44	.33–.59	*p* < .001	---
	Country^c^	---	---	*p* < .001	---
	- Germany	.09	.06–.14	*p* < .001	---
	- Hungary	.35	.26–.47	*p* < .001	---
	- Ireland	.24	.18–.32	*p* < .001	---
	Gender X Age Group	---	---	*p* < .001	---
	Gender X Country	---	---	*p* = .07	---
Parasuicidal pause	Male gender^a^	.55	.31–.96	*p* = .03	.165
	Age group^b^	---	---	*p* = .003	---
	- 30-45 years	1.07	.83–1.38	*p* = .61	---
	- >45 years	.7	.54–.92	*p* = .009	---
	Country^c^	---	---	*p* < .001	---
	- Germany	1.54	1.13–2.11	*p* = .006	---
	- Hungary	7.77	5.87–10.28	*p* < .001	---
	- Ireland	.81	.57–1.15	*p* = .24	---
	Gender X Age Group	---	---	*p* = .23	---
	Gender X Country	---	---	*p* = .179	---
Parasuicidal gesture	Male gender^a^	.61	.42–.88	*p* = .009	.098
	Age group^b^	---	---	*p* = .172	---
	- 30-45 years	.83	.67–1.03	*p* = .09	---
	- >45 years	.85	.68–1.05	*p* = .13	---
	Country^c^	---	---	*p* < .001	---
	- Germany	.67	.54–.84	*p* < .001	---
	- Hungary	.09	.054–.84	*p* < .001	---
	- Ireland	.99	.80–1.232	*p* = .95	---
	Gender X Age Group	---	---	*p* = .20	---
	Gender X Country	---	---	*p* = .009	---
Serious suicide attempt	Male gender^a^	1.93	1.42–2.62	*p* < .001	.071
	Age group^b^	---	---	*p* < .001	---
	- 30-45 years	1.14	0.95–1.36	*p* = .17	---
	- >45 years	1.88	1.56–2.26	*p* < .001	---
	Country^c^	---	---	*p* < .001	---
	- Germany	2.95	2.41–3.6	*p* < .001	---
	- Hungary	1.32	1.07–1.64	*p* = .01	---
	- Ireland	2.32	1.9–2.84	*p* < .001	---
	Gender X Age Group	---	---	*p* = .03	---
	Gender X Country	---	---	*p* = .02	---

*CI* confidence interval, *p* significance level, *R* ^*2*^ proportion of explained variance regarding the logistic regression model

^a^Reference category: female

^b^Reference category: Younger Age

^c^Reference category: Portugal

If a patient was male, the odds that he was rated as a SSA significantly increased (OR = 1.93; *p* < .001) while the odds for SP (OR = 0.55; *p* = .03) and SG (OR = 0.61; *p* = .009) significantly decreased. Compared to younger participants (< 30 years), the odds of people older than 45 years of age engaging in an act of DSH, or a SP significantly decreased (OR = 0.44; *p* < .001; OR = 0.7, *p* = .009) whereas corresponding odds for a SSA significantly increased (OR = 1.88; *p* < .001).

There was also a significant association between type of suicide intent and the variable “country” (*p* < .001).

Compared to Portugal, the odds of Germans, Hungarians and Irish suicide attempts being rated as a SSA (OR = 2.95; *p* < .001; OR = 1.32; *p* = .01; OR = 2.32; *p* < .001, respectively) significantly increased, while the odds for suicide attempts being rated as DSH (OR = 0.09; *p* < .001; OR = .35; *p* < .001; OR = .24; *p* < .001, respectively) significantly decreased.

Further to this, the analysis also revealed a significant interaction effect between gender and age group in the categories of DSH (*p* < .001) and SSA (*p* = .03), and also a significant interaction between gender and country in the SG (*p* = .009) and SSA (*p* = .02) category.

## Discussion

### General findings

The main aim of the current study was to examine gender differences in suicide intent in a large European cross-national sample, and to explore whether gender differences exist across age, country and suicide method. The results support the hypothesis that males would demonstrate a higher frequency of Serious Suicide Attempts (SSA) than females. In line with our other hypotheses, our results showed a significant gender difference between age groups for suicide intent, where in all age groups male suicide attempts were rated significantly more frequently as SSA compared to females. However, in the oldest age group, male suicide attempts were rated as Deliberate Self-Harm (DSH) significantly more times than female attempts. The hypothesis that male attempts would be rated as SSA more frequently than females within each country was supported in Portugal and Ireland, however, there were no significant differences in Germany and Hungary. Finally, our results confirmed the prediction that within the most utilised method of attempted suicide, in our case - intentional drug overdose, male suicide attempts would be rated as SSA more frequently than females.

The regression analyses revealed that suicide attempts of older people (>45 years) were more likely to be rated as a SSA compared to younger people, and younger people were more likely to be rated in the DSH and SP group than older people.

### Are there gender differences in intent?

The overall finding, that male attempts were rated as SSA more often than females, is in line with other studies that found females to have a less serious intent to die than males [15, 18, 31, 32] despite other findings illustrating no difference in suicide intent between males and females [6, 19, 33].

### Are gender differences in intent dependent on age?

The association between suicide intent and age, which was found in previous studies [14, 15, 34] was not supported in the current study. In contrast with the results of previous studies that reported that young females had a significantly higher level of suicide intent than young males [35], in the present study, the proportion of young males (<30 years) rated as SSA was significantly higher than the young females. Further to this, previous studies have reported the lower age of females at the time of suicide attempt as a general trend [22, 36, 37] which was not confirmed in this study.

### Are gender differences dependent on country?

In support of previous studies [38–40], a significant gender difference in suicidal intent across countries was revealed. Hungary, Ireland and Portugal reported significant differences between males and females in suicide intent, whereas no significant differences were reported for Germany. Current findings however contradict those of Hjelmeland et al. [31, 41] whose results of a cross-cultural study on suicide intent found that there were no gender differences in intent and country. Hjelmeland’s [41] findings however, support the current findings from Germany, which suggests that suicide intent is posited to be similar for both genders.

### Are gender differences in intent dependent on method of suicide attempt?

In terms of the association between type of suicide intent and gender among different suicide methods, results illustrated that for suicide intent, SSA was rated significantly more frequently in males than females in the most frequently used method of attempted suicide (intentional drug overdose, *N* = 3542, 67.9% of patients). This finding propounds that even within the same method of attempted suicide, in this case, intentional drug overdose, males show a stronger intent to die than females. This finding is in line with a recent study of over four thousand self-harm cases, which reported a significant association between higher estimated median suicide intent scores with male gender, self-poisoning, multiple methods of self-harm, use of gas, use of alcohol and dangerous methods of self-harm [42]. Thus, it can be inferred that irrespective of the method of self-harm, male suicide attempts tend to be more serious than female suicide attempts.

### Why are there gender differences in suicide intent?

The results presented above support previous evidence showing that males have a higher intent to die than females. This evokes questions as to why this would be the case. Some theorists contend that females attempt suicide earlier in the evolution of psychiatric morbidity than males, which might represent less of an intention to die, and more a desire to communicate distress or change their social environment [21, 22]. Another theory suggests that because males have a higher intent to die than females, females may be more reluctant to perform a SSA because it is considered ‘masculine’ [6]. These gender-specific beliefs and attitudes towards self-harm may contribute to the explanation of young men’s low rates of suicidal behaviour and their high rates of suicide mortality [43], however, more research needs to be conducted in this area in order to develop concrete theories to support prevention efforts.

### The value of and barriers to measuring suicide intent

The concept of intent is a critical component in the clinical appraisal of suicide attempts, as it distinguishes between acts of deliberate and accidental self-harm. Many studies have assessed the value of measuring suicide intent for screening purposes and for its use in assessing future suicide risk, as the type of suicide intent has been associated with future suicide attempts [44, 45]. Therefore, the measurement of suicide intent may be particularly useful in the assessment of short-term suicide risk [46].

There are several measures to assess suicide intent, where Beck’s Suicide Intent Scale represents the most widely used one [47], however, the Feuerlein Scale [30], the five-point ordinal scale developed by Dorpat and Boswell [48] and other intent assessment instruments highlight the variability in the empirical measurement, nomenclature and analysis of suicide intent, and this lack of consistency and standardisation impedes future research related to the measurement of suicide risk and outcome [13]. Moreover, notwithstanding the clinical importance of assessing suicide intent, emergency department personnel often do not document suicide intent at all [49], despite national guidelines and policy initiatives recommending that psychosocial assessments (which include measuring suicide intent) must be undertaken after every self-harm presentation [50]. It is recommended that a standardised measurement for suicide intent is implemented in clinical settings in order to develop and effectively manage the treatment of patients at risk of suicide.

### Strengths and weaknesses

Although care was taken in the systematic collection of the data in all participating countries, in some regions, data was collected by proxy as well as self-report. The judgement about the suicide intent of the presenting cases was made by clinical staff based on the available information on the suicide attempt, and therefore was not self-report, which is suggested to be a more reliable method of measurement. Furthermore, only part of the overall data on suicide attempts available from the OSPI-Europe database of suicide attempts contained information on suicide intent. This led to an overall reduced sample size when compared to the total number of cases. Finally, due to the complexity of suicide intent, categorizing suicide intent with the Feuerlein Scale may not be a sufficiently precise categorization to detect gender differences. A methodological strength of the current study is the sample size (*n* = 5212), which increased the validity and power of the findings. Furthermore, compared to other cross-national studies, there was no variation in the definition used for selecting the samples of patients to be included in the study and a standardised assessment instrument was also utilised.

### Implications for future research and practice

In the current study, the previously reported gender differences between males and females in suicide intent were confirmed. However, remaining ambiguities call for further studies to include the variable ‘gender’ when conducting research on suicide. In order to reach a better understanding of the association between gender and suicide intent, cross-national studies to confirm or reject the transferability of the present findings and studies exploring the reasons for varying levels of suicide intent between males and females is needed. Nevertheless, in consideration of studies reporting gender differences in suicide intent, it seems reasonable to tailor prevention and intervention strategies to target gender-specific aspects. For instance, it would be justified to design an awareness campaign specifically targeting men, as our findings confirm that male suicide attempts are in general more serious than female attempts. Furthermore, given that female suicide attempts are more likely to be rated as Parasuicidal Pauses and Parasuicidal Gestures, it would be clinical valuable to specifically target at-risk females with low-level psychoeducational intervention, in order to help them communicate their distress more effectively.

In terms of the implications for age, the outcomes of the present study support the notion that age may impact on the level of suicide intent. Therefore, it is recommended that considering age in the assessment of suicide attempts is an important factor in the study of suicidal behaviour, as our findings show that specific ages for each gender are deemed to be more “vulnerable” to different levels of suicide intent and should be the focus of suicide prevention strategies.

The differences between countries regarding the association between gender and type of suicide intent that is presented here expose potential cross-cultural differences, which may have possible implications for the transferability of interventions. Further to this, the outcome that cultural influences play an important role in the gender paradox of suicidal behaviour has significant implications for research and for public policy makers.

The other finding from the current study which shows that even within the most frequently used method of attempted suicide, males report a higher level of suicide intent than females, also has significant implications for prevention efforts. Mental health promotion initiatives, such as public health strategies, should target “at-risk” groups, which may contribute to reducing suicide in general, but also to narrowing the gender imbalance in suicidal behaviours.

## Conclusion

The findings presented here have clinical and practical implications which may guide future practice such as assisting national policy makers and health services in identifying vulnerable groups. In terms of assessment and intervention strategies, the use of a validated and reliable instrument to the assess level of suicide intent is likely to be beneficial to use in assessments in clinical practice to help guide and manage treatment strategies, and may also serve as a valuable basis to assess the future risk of the patient. Further insight into this area is the first step to understanding the meaning that these suicidal patients ascribe to their suicidal behaviour. Finally, our findings also shed some light on the gender paradox that exists in the incidence of suicide attempts and completed suicides by identifying the complex differences in suicide intent between males and females who attempt suicide. Considering the gender differences in suicidal intent highlighted by the study, targeted preventive interventions are warranted.

## Abbreviations

DSH: Non-habitual deliberate self-harm
EAAD: European alliance against depression
OSPI Europe: Optimising Suicide Prevention Programmes and their Implementation in Europe
SG: Parasuicidal gesture
SP: Parasuicidal pause
SSA: Serious suicide attempt
